# Implications of No Tail Docking on Performance, Health, and Behavior of Pigs Raised Under Commercial Conditions in Brazil

**DOI:** 10.3390/ani15091308

**Published:** 2025-04-30

**Authors:** Juliana Cristina Rego Ribas, Joseph Kaled Grajales-Cedeño, Isadora Gianeis, Vivian S. Sobral, Mateus José Rodrigues Paranhos da Costa

**Affiliations:** 1 Departamento de Boas Práticas e Bem-Estar Animal, Agroceres PIC, Rio Claro 13502-741, RJ, Brazil; 2 Programa de Pós-Graduação em Ciência Animal, Faculdade de Ciências Agrárias e Veterinárias, UNESP, Jaboticabal 14884-900, SP, Brazil; joseph.kaled@unesp.br (J.K.G.-C.);; 3 Grupo de Estudos e Pesquisas em Etologia e Ecologia Animal (ETCO), Faculdade de Ciências Agrárias e Veterinárias, UNESP, Jaboticabal 14884-900, SP, Brazil; 4 Facultad de Ciencias Agropecuarias, Departamento de Zootecnia, Universidad de Panamá, Chiriquí 7096, Panama; 5 Departamento de Zootecnia, Faculdade de Ciências Agrárias e Veterinárias, UNESP, Jaboticabal 14884-900, SP, Brazil; 6 CNPq, Conselho Nacional de Desenvolvimento Científico e Tecnológico, Brasília 71605-001, DF, Brazil

**Keywords:** animal welfare, branched chains, environmental enrichment, fear, piglets, stress

## Abstract

**Simple Summary:**

This study evaluated the effects of no tail docking on the performance, health, and behavior of piglets under commercial conditions in Brazil. A total of 768 piglets were divided into two groups: tail-docked (DT) and non-tail-docked (NDT). Both groups received standard environmental enrichment, with additional enrichment provided in cases of tail bite. The NDT piglets exhibited a higher tendency for tail biting and severe lesions, particularly during the nursery phase, but also demonstrated more exploratory behavior. In the finishing phase, tail biting was only observed in the NDT group. Fear responses to humans vary depending on whether additional enrichment is required or not. Despite the presence of tail lesions, productivity was not affected. Overall, managing piglets with intact tails posed welfare challenges, whereas docking the final third of the tail combined with best management practices reduced negative effects on piglet welfare.

**Abstract:**

This study aimed to evaluate the effects of no tail docking on the performance, health, and behavior of piglets raised under commercial conditions in Brazil. The study included 768 weaned piglets from the Pietrain synthetic line, randomly divided into two groups: DT = the final third part of the tail-docked (n = 384) and NTD = non-tail-docked (n = 384). Tail docking was performed on day two using an electrocautery clipper for piglets from the DT group, and both groups were subjected to standard environmental enrichment with branched chains. In cases of tail biting, a contingency plan was adopted to mitigate this problem by enriching the pen with a sisal rope. Behavioral measurements were performed using scan sampling. Tail biting, reactivity to humans, and health were assessed using a methodology adapted from the Welfare Quality Protocol^®^. The piglets were weighed at 140 days of age and inspected according to the parameters established by the Pig Genealogical Registration Service to be used as reproduction animals. The off-test rate was calculated based on the total number of piglets approved for animal use relative to the total number evaluated. During the nursery stage, the NDT piglets showed a trend toward significance (*p* = 0.07) toward a higher occurrence of tail biting than the DT piglets and exhibited a higher incidence of severe lesions. They also engaged more frequently (*p* < 0.05) in exploratory behavior, interacting with branched chains and sisal rope, than the DT piglets. During the finishing phase, tail biting was observed only in the NDT piglets (*p* = 0.001). The NDT piglets that did not require the contingency plan exhibited lower fear responses (*p* = 0.02) during human interactions in the nursery phase than the DT piglets. Conversely, the NDT piglets that required a contingency plan showed higher fear levels (*p* < 0.001). Productivity performance was not affected (*p* > 0.05), and new cases of tail biting ceased after the contingency plan was implemented. The number of animals that died or were removed did not differ between the treatments (*p* > 0.05). In conclusion, managing piglets with intact tails on commercial farms presents a significant welfare challenge. By contrast, docking the final third of the tail, in accordance with regulations, was associated with fewer negative welfare outcomes, even when best management practices were applied.

## 1. Introduction

Pork consumption trends have been influenced by several factors, including cultural preferences, health perceptions, access to product information, and the way pork is presented and sold [1]. Additionally, consumer awareness of sustainable practices in animal production, their impact on CO_2_ emissions, and animal welfare concerns have become increasingly relevant to pork consumption choices [2]. Understanding these trends and adopting appropriate measures to meet market demands and consumer expectations are essential for promoting responsible and sustainable pork consumption [3]. 

Animal welfare is intrinsically linked to the 17 UN Sustainable Development Goals [4]. Well-treated animals are healthier and more productive, and can contribute to reducing poverty and social inequalities, maintaining ecological balance, and promoting efficient use of resources. Owing to growing consumer concerns about pig welfare, legislation addressing this issue has increased, leading various countries to establish minimum standards for pig farming. For example, Directive 2008/120/EC of the European Council (EU, [5]) regulates the commercial breeding of pigs across all European Union member states. Following the publication of this directive, several regulations and guidelines have been introduced in other countries, including Canada, South Africa, Australia, and some states in the United States. Although these regulations vary in detail, they generally mandate more space in intensive farming systems and restrict stressful and painful management procedures such as tail docking, caudectomy, teeth trimming, and surgical castration [6].

On 16 December 2020, Brazil introduced Normative Instruction No. 113/2020, which also addressed regulations on raising pigs in commercial farm units [7]. Among its provisions, Article 36 addresses tail docking, stipulating that it is permitted only within the first three days of life, using an electrocautery clipper, or if performed later, must be performed with anesthesia and analgesia. Additionally, only the final third of the tail was removed. Chapter VI of the same normative mandates the implementation of environmental enrichment.

Raising pigs with intact tails to promote animal welfare and meet consumer demands poses a challenge for pig farmers as it increases the risk of tail biting behavior [6,8]. This behavior is influenced by multiple factors, including genetic and environmental conditions such as air temperature, stocking density, access to food and water, availability of enrichment material, and tail length [3]. Several studies comparing different tail lengths in pigs have found a higher prevalence of tail biting in pigs with intact tails than in those with part of the tail docked [3,9,10,11]. Furthermore, the authors observed that tail biting negatively affects animal welfare, reduces performance, and increases mortality rates or carcass condemnation. This situation presents a dilemma: to dock or not to dock the pig’s tail? It is essential to find a solution that allows pigs to retain their tails without increasing the risk of tail biting. This study aimed to assess the effect of maintaining intact tails in pigs raised under commercial farming conditions in Brazil. Our hypothesis was that maintaining intact tails in pigs raised under commercial conditions in Brazil does not negatively affect their welfare when appropriate management practices are implemented.

## 2. Materials and Methods

The study was carried out on a commercial farm with the capacity to house 1500 sows in Minas Gerais state, Brazil, after approval by the Ethics Committee on the Use of Animals of the Faculty of Agricultural and Veterinary Sciences, São Paulo State University, Jaboticabal, SP, Brazil (Protocol Number 3152-21).

### 2.1. Animals and Facilities 

A total of 784 piglets from the synthetic Pietrain line were randomly assigned to two experimental groups: NDT, piglets with intact tails (NTD, n = 384), and TD, piglets with docked tails (TD, n = 384), in which only the final third of the tail was removed on day two using an electrocautery clipper. After the procedure, Terracotril^®^ (Zoetis, Parsippany, NJ, USA), an antibiotic and anti-inflammatory spray, was used to mitigate complications from the procedure, and piglets from both groups were housed in 24 pens (~16 piglets per pen, grouped by sex), ensuring standardized density, free access to water and feed, and the provision of environmental enrichment in compliance with MAPA Normative Instruction No. 113/2020 [7].

At 24 days of age, the piglets were housed in the nursery room in pens measuring 2.15 × 1.95 m, with an 80% metal slated floor and 20% cement compact in front of the feeder. Each pen contained two nipples for access to water and half of a Cristal Spring^®^ deposit feeder (Crystal Springs Hog 1950 Equipment, Sta. Agathe, MB, Canada) for ad libitum feeding. The piglets had access to a 3.63 cm/animal feeding space. The stocking density during this phase was 3.7 animals/m^2^ (0.27 m^2^/animal), grouped by sex. The groups remained together during the nursery and rearing and finishing phases.

**Figure 1:**
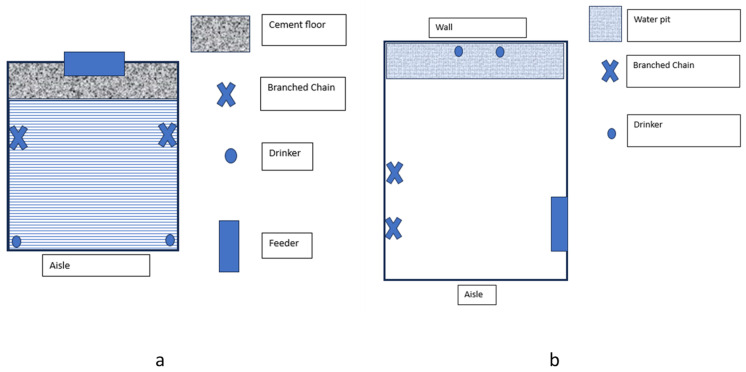
Schematic representation of the nursery (**a**) and rearing/finishing (**b**) pens.

The nursery rooms (Figure 1a) had a mechanical ventilation system with two groups of evaporative panels and five extractors using a Viper Touch^®^ controller (Big Dutchman Brazil, Araraquara, SP, Brazil). During the nursery phase, three rooms and 48 pens were used. The adopted thermal regime followed the temperature curve according to the Agroceres PIC Growing Guide [12], ranging from 23 °C (after twenty-eight days of housing) to 27 °C (at weaning). Each pen had infrared light to promote a better microclimate for the piglets during the first week. The brightness of the room was 150 lx, with lightning occurring from 7:00 a.m. to 8:00 p.m. The piglets remained in the nursery for an average of 41 d. 

On average, at 64 days, the piglets were transferred to the rearing/finishing rooms (Figure 1b), maintaining the same groupings (excluding the piglets that died or were removed after selection). This phase used three rooms, each containing 32 pens (5.17 × 3.0 m, with a stocking density of 0.97 m^2^/animal). The pens had a compact floor with a 30 cm water pit at the end. Each pen was equipped with two hanging nipple drinkers and had access to half of a Cristal Spring^®^ deposit feeder (Crystal Springs Hog 1950 Equipment, Sta Agathe, MB, Canada), providing 4.76 a of feeding space. The environment was naturally ventilated, with lighting maintained at 150 lx, following a natural photoperiod. In both phases, the pen was considered the experimental unit. 

The piglets were identified individually using ear tags. Dead animals were removed from the pens, and their data were recorded. The animals received identical diets throughout the experimental period, and feed was provided ad libitum according to the nutritional requirements of each phase [13].

### 2.2. Experimental Design

The piglets were randomly assigned to two treatments: NTD = no tail docked, piglets with intact tail (n = 384, 24 pens), and TD (piglets with the final third part of tail docked on the second day of life (n = 384, 24 pens). Tail docking was performed using a clipper electrocautery MS Smarter Tail Docker (MS Schippers, Hapert, The Netherlands), followed by the application of an antiseptic spray with a topical anti-inflammatory agent. The procedure ensured that two-thirds of the tail remained intact, in accordance with MAPA Normative Instruction N°113/2020 [7]. The procedure was performed in accordance with the Brazilian Swine Welfare Standard (IN 113/2020), which allows tail docking without anesthesia in the first three days of life. 

In the rearing/finishing phase, a reduced number of piglets (NTD = 351 and TD = 357) were assessed due to mortality and removal. The distribution of piglets in the nursery and rearing/finishing phases according to the treatments, number of pens, number of piglets per pen, and total number of piglets are shown in Table 1.

In all the pens during both the nursery and rearing/finishing phases, a standardized environmental enrichment protocol was implemented by installing branched chains, as proposed by Bracke [14], at a ratio of 1 point per 12 piglets. The chains were 5 mm thick, with a main axis of 60 cm in length and three filaments of 15 cm each spaced 10 cm apart. They were installed at the animal’s eye level and each pen was equipped with two sets of chains.

A standardized contingency plan, as defined by Chou et al. [15], was implemented when tail biting was observed. This plan included the immediate treatment of injured animals with a healing spray, the addition of extra environmental enrichment points using sisal rope at a ratio of one point for five piglets per pen, and, when necessary, the transfer of the injured individuals to a hospital pen. The sisal rope was 2 mm thick and 1.20 m long in both the nursery and finishing phases, with two filaments of equal length. Three ropes were placed near the end of each pen.

### 2.3. Tracking Environmental Conditions

The air temperature and humidity were recorded using Log Tag^®^ data loggers (model TRIX-8, LogTag, Lafayette, NJ, USA) programmed for sequential records with an interval of 1 h between them. The devices were positioned strategically in the central aisle of the experimental rooms. The mean, minimum, and maximum values of the air temperature (°C) and relative air humidity (%) recorded during the nursery and rearing/finishing phases are shown in Table 2.

### 2.4. Performance Assessment

The body weights of the piglets were recorded one week after birth, at the end of the nursery phase (63 days), and at the end of the rearing and finishing phases (140 days). Average daily gain (ADG) was calculated based on the initial and final body weights, considering the time interval (in days) between the measurements.

The zootechnical inspection parameters were recorded following the recommendations of ABCS [16]. The selection rate was calculated as the total number of approved animals divided by the total number of evaluated animals. In addition, the reasons for non-selection were recorded, which led to the removal of certain piglets from the experiment.

### 2.5. Piglets’ Reactivity and Other Behavior Assessment

Piglet reactivity to humans was assessed in both phases by introducing a familiar person to the pen. The person walked around the pen, paused for 30 s, and then returned in the opposite direction without interacting with the animals. During the second pass, the piglets’ reactions were scored as follows: 1 = the piglets approached the person without displaying panic, or only a few showed mild signs of fear; 2 = more than 60% of the animals exhibited signs of panic or fear, such as cowering at the bottom of the pen, running away, or vocalizing.

This method was selected to replicate real-life interactions between farm workers and piglets. The assessment was conducted weekly, always in the morning, using a randomly selected subsample of 12 pens representing 20% of the total experimental pens. 

Other behaviors were recorded using the scan sampling method, considering the behavioral categories described in Table 3 [17]. The number of animals engaged in each activity was recorded for each sampling point. Data collection began on the second day after housing the piglets and was conducted three times per week, twice a day (09:00 and 14:00 h). The behavioral data were recorded by a trained observer.

### 2.6. Deaths, Tail Biting, and Other Health Indicators

The total number of deaths and removals was counted at the end of the nursery phase. The piglets with low indices, which were no longer suitable for genetic improvement programs, were sent to another farm to be raised as fattening pigs, and were, therefore, removed from the experiment. 

Tail biting was assessed daily by recording the bite injuries in the morning. Bite injuries were scored as follows: 0 = absent, 1 = mild (slight abrasion or oral contact mark), and 2 = severe (bleeding or removal of part of the tail). Severe tail injuries were recorded and handlers were required to initiate the contingency plan as described previously.

Other health indicators were assessed weekly in all the pens following the Welfare Quality^®^ Protocol [17]. The piglets’ health conditions were scored individually based on ten indicators: animal hygiene, signs of thermal discomfort, body wounds, diarrhea, laminitis, local infections, hernia, prolapse, body condition, and calluses or bursae.

### 2.7. Statistical Analysis

Statistical analyses were performed using the R software (version 4.3.0,Viena, Austria, RStudio, Inc.) in the RStudio integrated development environment. For all the statistical tests, differences were considered statistically significant when *p* ≤ 0.05, and trends were considered significant when *p* > 0.05 and ≤ 0.10.

Piglet weights at the first week and end of the nursery phase were analyzed using a generalized linear model, with treatment (TD and NTD), sex (male and female), and their interactions as fixed effects, and birth weight as a covariate. Weight at the end of the rearing/finishing phase was analyzed using a mixed linear model, considering treatment (TD and NTD), sex (male and female), and their interactions as fixed effects; pen as a random effect; and the age at which the animals were selected as covariates.

The percentage of animals according to the reactivity scores per treatment was analyzed using the chi-square test (χ^2^), with Yates or Fisher’s exact test correction applied when necessary, considering that each pen required the implementation of a contingency plan. 

Other behavioral categories were analyzed using mixed linear models, considering the treatment (TD and NTD), weeks of assessment (1–5), sex (male and female), and their interactions as fixed effects, and pen as a random effect. Since sex was not significant for any of the behavioral categories, it was excluded from the final statistical model. The behavioral categories “sitting or standing”, “positive social interaction”, and “interaction with the sisal rope” in both phases required logarithmic transformation to meet the assumptions of normal distribution of residuals (normal quantile–quantile plot, histogram, and Anderson–Darling test). The results of these variables are presented on the original scale. In the nursery phase, the negative behavioral categories (“tail, ear, and flank biting”) did not follow a normal distribution even after transformation; therefore, they were expressed as percentages of occurrence. 

The chi-square test was used to assess the effects of treatment on the incidence of tail biting during the nursery and rearing/finishing phases. The occurrence of tail biting was reported according to the piglet age (weeks). The percentages of pens with contingency plan implementation are shown according to the treatment (TD and NTD) and assessment phases (the nursery and rearing/finishing phases).

The total number of deaths and animals that needed to be removed at the end of the nursery phase was analyzed using the chi-square test. Regarding health indicators, there were no reports of disease conditions during the project.

The best model fit for the adopted models (mixed linear models and generalized linear models) was determined using the “step-up” procedure based on the Akaike Information Criterion (AIC) and the Bayesian Information Criterion (BIC). Multiple comparisons for all the models were performed using Tukey’s test to compare the adjusted means (function “emmeans” of the “emmeans” package) [18]. 

Outliers were duly identified and maintained as they were considered individual variations. Box plots and bar graphs were created as appropriate, representing information on the original scale of the variables. 

## 3. Results

### 3.1. Performance Indicators

No significant differences in body weight were observed during the first week of life or at the end of the nursery phase (*p* > 0.05; Table 4). However, in the rearing/finishing phase, a statistically significant difference in body weight and ADG was found based on sex (*p* < 0.05), with males consistently showing a higher body weight and ADG than females (Table 4). There were no significant differences between treatments in the selection rate, with TD = 60% and NTD = 64% (χ^2^ = 0.24; *p* = 0.24).

### 3.2. Piglets’ Reactivity and Other Behavior

During the nursery phase, a higher percentage of the NTD piglets that did not require the implementation of a contingency plan were less reactive than the TD piglets (*p* < 0.05; Table 5). Conversely, when considering the pens that required a contingency plan, a higher percentage of the TD piglets were less reactive. In the rearing/finishing phase, no significant variations in reactivity scores were observed between treatments regardless of contingency plan implementation (Table 5).

The percentages of animals displaying resting, exploring the pen, interacting with the branched chain and sisal rope, engaging in positive social interaction, and exhibiting other behaviors were significantly affected by the interaction between the treatment and week of assessment (*p* < 0.05). In the first and fifth weeks of the nursery phase, a higher percentage of the NTD piglets explored the pen and displayed other behaviors than the TD piglets. Additionally, in the fourth week of the same phase, the NDT piglets subjected to the contingency plan interacted more with the sisal rope than the TD piglets. Significant variations (*p* < 0.05) were observed across the weeks for both treatments (Table 6). 

Sitting or standing behavior, as well as the negative behaviors assessed, did not significantly differ between treatments (*p* > 0.05) or between the treatment and assessment weeks (*p* > 0.05). Furthermore, the treatment did not significantly affect any behavior assessed during the rearing/finishing phase (*p* > 0.05).

### 3.3. Results of Deaths, Tail Biting, and Other Health Indicators

The percentage of deaths or removal from the study at the end of the nursery phase for the NDT and TD piglets was 8.59% and 7.03%, respectively (χ^2^ = 0.02; *p* = 0.88). 

**Figure 2:**
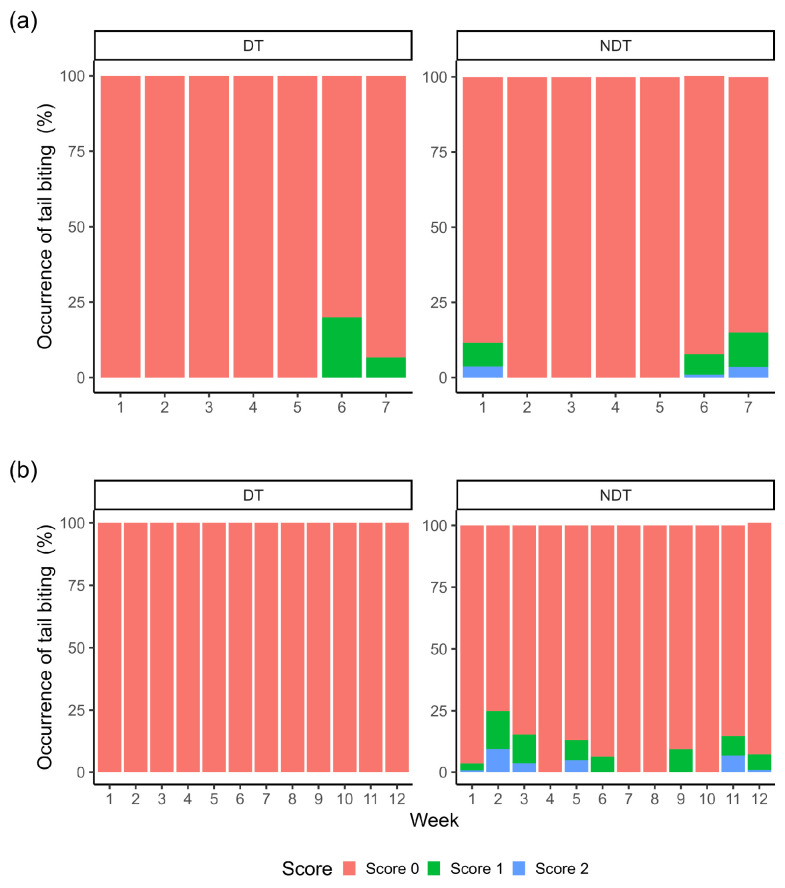
Percentages of piglets showing signs of tail biting throughout the assessment weeks during the nursery (**a**) and rearing/finishing (**b**) phases according to the treatment (TD and NTD).

There was a tendency (*p* = 0.08) for the treatment to affect the percentage of bite scores recorded during the nursery phase. In contrast, during the finishing phase, tail biting injuries were recorded only in the NDT piglets (Table 7 and Figure 2). During the nursery phase, tail biting injuries occurred more frequently in weeks 1, 6, and 7 for the NTD piglets and in week 7 for the TD piglets. Only the NTD piglets received a score of 2 for tail injuries, which implied bleeding and the removal of the tail parts (2.45% and 4.05% for the nursery and rearing/finishing phases, respectively).

There was a difference between treatments regarding the need to implement the contingency plan during the nursery phase (*p* < 0.001), a considerable proportion of pens housing the NTD piglets (14 out of 28, 50%) required a contingency plan compared to only a few (2 out of 28, 7%) of those housing the TD piglets. The difference remained evident during the rearing/finishing phase (*p* < 0.001), with a higher proportion of pens with the NTD piglets (13 out of 28, 46%) requiring a contingency plan, whereas none (0 out of 28, 0%) of the TD piglets needed intervention. The percentage of pens in which the contingency plan was implemented is listed in Table 8.

It is important to highlight that piglet removal due to tail biting did not occur during the nursery phase. However, during the rearing/finishing phase, removal was necessary for both groups, with 4 out of the 351 (1.13%) NDT piglets and 1 out of the 357 (0.28%) TD piglets being removed. No other health problems were recorded during either phase of the study.

## 4. Discussion

In this study, we hypothesized that maintaining pigs with intact tails under commercial conditions in Brazil would not compromise animal welfare, provided that appropriate management practices were applied. However, our findings suggest that tail preservation can have a detrimental impact on welfare even when the current regulatory standards are met. Although the implementation of a contingency plan may partially alleviate negative consequences, it does not eliminate the substantial initial risk associated with keeping pigs with intact tails.

These results offer important insights into the effects of tail preservation on pig performance, health, and behavior under Brazilian commercial farming conditions. Notably, the piglets with non-docked tails (NTD) showed a higher tendency for severe tail biting incidents than those with docked tails (TD), both during the nursery and growing-finishing phases. These findings are consistent with previous reports by Hunter et al. [19,20] and Wallgren et al. [11], who observed a higher prevalence of severe tail lesions (2–12%) in pigs with intact tails, those with a lower incidence (approximately 3%) in the docked pigs.

According to Chou et al. [15], tail biting results from multifactorial factors, meaning that a specific combination of environmental conditions creates unique characteristics that influence the likelihood of tail biting outbreaks. Consequently, solving this problem is not straightforward, leading farmers and technicians to adopt tail docking routinely.

Contrary to the European Food Safety Authority (EFSA) [21], which suggests that tail biting cannot be effectively controlled solely through facility adaptations and management strategies, our study presents different findings. The stocking densities and other pig-rearing conditions used in this study were similar to those described in previous studies, yet the results differed. A key distinction is that the environmental enrichment used in this study was non-destructive, potentially fulfilling the animals’ behavioral needs and reducing the percentage of pigs with severe tail injuries across both evaluation phases. These findings align with a study conducted by Valros & Heinonen [6] in Finland (where tail docking is prohibited); the authors found that only 2.3% of the pigs exhibited tail biting injuries in a dataset of approximately 1.6 million slaughtered pigs. The authors attributed the low occurrence to factors such as lower stocking density (~0.8 m^2^ per pig) and the provision of manipulable materials.

Mild tail biting injuries (score 1), characterized by slight abrasions or oral contact marks, are rarely discussed in the literature, likely because of their lower impact on secondary injuries that can affect animal performance and health. However, these injuries are also relevant indicators of poor welfare. Our results showed a higher frequency of score one tail biting injuries during the first and last two weeks of the nursery phase. This pattern suggests a stress response to weaning, a period characterized by the increased oral manipulation of penmates and surroundings, as noted by Ipema et al. [22], particularly in environments with minimal or no enrichment. Another possible explanation for the higher frequency of mild injuries in TD piglets during the nursery phase (15% vs. 8%) is the long-term stress induced by tail docking. Studies [23,24,25] have suggested that this painful practice has prolonged behavioral effects.

Our findings also indicated an increase in tail biting injuries in both the TD and NTD groups at the end of the nursery phase, coinciding with the expected increase in weight gain by the piglets and when there was a consequent reduction in the available space per pig. This highlights the need to reassess the proposed stocking density regulations for this phase in Brazil and European countries.

Furthermore, human–animal interaction assessment in the nursery phase revealed that the NTD piglets exhibited lower levels of fear when interacting with humans than the TD piglets. These results corroborate the findings of Sutherland et al. [25], who suggested that long-term pain and stress caused by tail docking contribute to heightened fear responses in pigs. Similarly, Hemsworth et al. [26] reported that pigs exposed to positive human interactions were generally less fearful and more willing to approach handlers. Our results reinforce the hypothesis that the painful experience of tail docking negatively affects interactions between pigs and humans.

However, when the contingency plan was implemented, the NTD piglets with tail injuries exhibited greater fear than the TD piglets did, further emphasizing the impact of pain on pig behavior. According to Valros et al. [27], pigs experiencing chronic tail biting injuries show signs of hypercortisolism, which is indicative of chronic stress. Similarly, Munsterhjelm et al. [28] reported increased adrenal gland size and elevated cortisol concentrations in pigs with chronic tail biting.

During the finishing phase, tail biting injuries were recorded only in the NTD piglets, with grade 1 and 2 injuries worsening during the housing period. The observed decline in tail biting incidents after weeks 2, 5, and 11 likely resulted from the implementation of the contingency plan, as recommended by Chou et al. [15]. These results emphasize the importance of having a contingency plan in place to manage tail biting in pigs. Notably, 50% and 40% of the NTD piglets’ pens required contingency measures during the nursery and finishing phases, respectively. No piglets were removed because of the tail biting injuries during the nursery phase, and only 0.95% were removed during the rearing/finishing phase, demonstrating the contingency plan’s effectiveness in controlling this issue.

During the nursery phase, the NTD piglets engaged more frequently in positive behaviors, such as exploration and interaction with branched chains and sisal rope, compared to the TD piglets. This difference may be attributed to the absence of chronic pain in the NDT piglets, which is expected to be present in TD ones [29]. However, no significant behavioral differences between treatments were observed during the rearing/finishing phase, likely because of the increased resting behavior associated with greater body weight [30]. This suggests that the effect of treatment is more pronounced during the nursery phase, highlighting the importance of evaluating the negative consequences of tail docking at specific developmental stages.

Regarding health indicators, no issues other than tail injury were recorded throughout the study. Additionally, no significant differences in the approval status of animals for breeding were observed between treatments. In terms of death or removal, the NTD group presented a high percentage of pigs that were out of the process (8.59% NTD vs. 7.03% TD), and these results were affected by the presence of severe tail biting lesions in the NTD group, as reported by Zhou et al. [31]. These lesions directly affect the number of animals viable for slaughter in the production chain, directly affecting the 17 UN Sustainable Development Goals [4]. No treatment effects were identified in terms of performance, which differs from the results reported by Zhou et al. [31] and Sutherland et al. [24], who reported reduced performance in pigs with tail biting injuries. 

The contingency plan proved effective in controlling tail biting and preventing the progression of injuries, thereby demonstrating its value as a management tool. Our findings suggest that maintaining intact tails may negatively affect piglet behavior during the nursery phase, particularly in stressful situations. However, aversive practices, such as tail docking, can harm human–animal interactions, which are essential for ensuring good welfare conditions for piglets. According to Valros & Heinonen [6], tail docking causes pain in 100% of pigs, whereas an intact tail results in pain only in affected individuals. The latter is considered more intense owing to the prolonged nature and intensity of tail biting [6]. In addition, in cases of tail biting outbreaks, the mental well-being of farm workers must also be considered, given the challenges associated with managing and treating injured animals.

The results underscore the importance of thorough research before transitioning away from tail docking, as the incidence of tail biting and associated injuries remains high in non-docked pigs, even when regulatory standards are followed.

## 5. Conclusions

Raising pigs with intact tails on Brazilian commercial farms, even when complying with MAPA Normative Instruction No. 113/2020 standards, poses a risk to animal welfare, as highlighted by the high prevalence of severe tail biting lesions in the nursery and finishing phases. One way to mitigate this risk of tail lesions is through the implementation of a contingency plan at the first sign of tail biting incidents. A contingency plan effectively prevents the progression of tail injuries and should be considered a crucial management strategy.

## Figures and Tables

**Table 1:** Number of pens, piglets per pen, and total number of piglets according to the weeks of assessment and treatments during the nursery and rearing/finishing phases.

Weeks	Treatments	Pens	Piglets per Pen	Total of Piglets
Nursery phase				
1	NTD	9	16	144
1	TD	9	16	144
2	NTD	9	16	144
2	TD	9	16	144
3	NTD	6	16	96
3	TD	6	16	96
Rearing/finishing phase				
1	NTD	9	15	132
1	TD	9	14	126
2	NTD	9	15	135
2	TD	9	16	141
3	NTD	6	14	84
3	TD	6	15	90

**Table 2:** Means, and minimum and maximum values of air temperature (°C) and relative air humidity (%) according to the nursery and rearing/finishing phases.

Air Temperature (°C)			Relative Air Humidity (%)		
Mean	Minimum	Maximum	Mean	Minimum	Maximum
Nursery phase					
24.63	22.32	27.61	72.24	57.60	83.20
Rearing/finishing phase					
21.15	15.55	27.73	61.65	49.70	80.80

**Table 3:** Behavioral categories of piglets assessed during the nursery and rearing/finishing phases.

Behavioral Categories	Description
Sitting or standing (SP)	Piglet supports the hindquarters or the four limbs on the floor, respectively, without performing any activity.
Resting (Rep)	Piglet is lying on the side or ventral position.
Exploring the pen (EX)	Piglet explores the pen, licking, biting, and smelling the pen’s structures.
Positive social interaction (IP)	Piglet sniffs, nuzzles, gently touches, or plays with another animal without triggering aggressive reactions on the part of the other individual.
Negative social interaction (IN)	Piglet shows aggressive behavior, fighting, mounting, headbutting, sucking the navel, or performing social behavior that bothers another animal.
Interaction with the branched chains (IC)	Piglet touches with its snout or bites the branched chains.
Interaction with the sisal rope (IO)	Piglet touches with its snout or bites the sisal rope.
Tail biting	Piglet performs oral manipulation of the tail of another animal.
Other behavior (OB)	Drinking, eating, moving around, urinating, and defecating.

**Table 4:** Adjusted means ± standard errors of average daily gain and body weight (kg) recorded during the first week in the nursery room and at the end of nursery and rearing/finishing phases, according to treatments (TD and NTD) and sex (male and female). Different letters indicate statistical significance between the means (*p* < 0.05).

	Treatment		Sex	
	TD	NTD	Male	Female
Average daily gain (g/d)	0.775 ± 0.065	0.782 ± 0.067	0.789 ± 0.064 ^a^	0.769 ± 0.069 ^b^
Body weight (kg) in the 1st week of life	2.50 ± 0.02	2.49 ± 0.02	2.50 ± 0.02	2.49 ± 0.02
Body weight (kg) at the end of nursery phase	24.6 ± 0.43	25.0 ± 0.43	25.0 ± 0.41	24.6 ± 0.45
Body weight (kg) at the end of rearing/finishing phase	109.0 ± 0.92	110.0 ± 0.94	111.0 ± 0.91 ^a^	108.0 ± 0.97 ^b^

**Table 5:** Percentages of piglets according to reactivity scores (1 = piglets approached the person without displaying panic, or only a few showed mild signs of fear; 2 = more than 60% of the animals exhibited signs of panic or fear, such as cowering at the bottom of the pen, running away, or vocalizing) based on treatment (TD = tail docked and NTD = no tail docking) and the need for contingency plan implementation during nursery and rearing/finishing phases.

Reactivity Score	Treatment		χ^2^ _(df)_	Fisher Exact Test	*p*-Value
	TD (%)	NTD (%)			
Nursery phase without contingency plan					
1	41.46	58.70	5.27 (1)	-	0.02
2	58.54	41.30			
Nursery phase with contingency plan					
1	100.00	55.56	59.59 (1)	-	<0.001
2	0.00	44.44			
Rearing/finishing phase without contingency plan					
1	96.25	98.39	-	-	0.68
2	3.75	1.61			
Rearing/finishing with contingency plan					
1	100.00	97.50	-	-	0.49
2	0.00	2.50			

**Table 6:** Adjusted means (±standard errors) of the percentage of the piglets displaying resting, exploring the pen, interacting with the branched chains and sisal rope, engaging in positive social interaction, and exhibiting other behaviors during the nursery phase according to the interaction between the treatment and week of assessment. The letters indicate statistical differences among the means (*p* < 0.05).

Treatment	Week of Assessment				
	1	2	3	4	5
Resting					
TD	76.2 ± 2.66 ^a^	59.9 ± 2.37 ^cde^	52.2 ± 1.66 ^f^	61.8 ± 1.88 ^cde^	73.3 ± 2.49 ^ab^
NTD	66.9 ± 2.56 ^abc^	56.6 ± 2.20 ^def^	54.5 ± 1.67 ^ef^	56.8 ± 1.88 ^def^	64.1 ± 2.56 ^bcd^
Exploring the pen					
TD	8.14 ± 1.65 ^cd^	14.41 ± 1.47 ^ab^	16.04 ± 1.05 ^a^	9.92 ± 1.18 ^bcd^	7.90 ± 1.55 ^cd^
NTD	16.72 ± 1.59 ^a^	14.16 ± 1.37 ^abc^	18.63 ± 1.05 ^a^	11.37 ± 1.18 ^bcd^	7.18 ± 1.59 ^d^
Interaction with the branched chain					
TD	2.14 ± 0.82 ^ef^	3.03 ± 0.75 ^bcde^	4.75 ± 0.59 ^ce^	6.16 ± 0.64 ^a^	3.34 ± 0.77 ^bcde^
NTD	3.50 ± 0.79 ^abcde^	4.34 ± 0.71 ^abcde^	2.80 ± 0.59 ^abc^	4.85 ± 0.64 ^abd^	3.09 ± 0.79 ^abcde^
Interaction with the sisal rope					
DT	0 ^bc^	0.08 ± 0.83 ^bc^	0.58 ± 0.68 ^bc^	1.57 ± 0.72 ^bc^	1.35 ± 0.87 ^bc^
NTD	0.19 ± 0.90 ^c^	3.37 ± 0.79 ^ab^	3.65 ± 0.68 ^ab^	5.21 ± 0.72 ^a^	2.44 ± 0.88 ^b^
Positive social interaction					
DT	0.56 ± 0.28 ^ab^	0.29 ± 0.24 ^ab^	0.08 ± 0.15 ^b^	0.18 ± 0.18 ^b^	0.08 ± 0.26 ^b^
NDT	0.41 ± 0.27 ^ab^	1.28 ± 0.22 ^a^	0.18 ± 0.15 ^b^	0.10 ± 0.18 ^b^	0 ^b^
Other behaviors					
TD	11.2 ± 1.34 ^cd^	15.8 ± 1.16 ^bc^	21.5 ± 0.69 ^a^	19.5 ± 0.85 ^ab^	13.4 ± 1.24 ^cd^
NTD	10.1 ± 1.28 ^d^	14.4 ± 1.06 ^cd^	20.9 ± 0.69 ^a^	21.1 ± 0.85 ^a^	21.0 ± 1.28 ^ab^

**Table 7:** Mean percentages of bites scores (0 = absent, 1 = mild (slight abrasion or oral contact mark), and 2 = severe (bleeding or part of the tail was removed) observed during the nursery and rearing/finishing phases, according to the treatment (TD and NTD).

Tail Biting Score	Treatment		χ^2^ (df)	*p*-Value
	TD (%)	NTD (%)		
Nursery phase				
Score 0	84.44	89.67	5.16 (2)	0.08
Score 1	15.56	8.03		
Score 2	0.00	2.45		
Rearing/finishing phase				
Score 0	100	87.89	13.06 (2)	0.001
Score 1	0	8.23		
Score 2	0	4.05		

**Table 8:** Total number of pens, and number and percentage (%) of pens that required the contingency plan implementation, as well added number of piglets evaluated and removed from pens due to severe tail biting injuries according to treatments (TD and NTD) during the nursery and rearing/finishing phases.

Phase	Treatment	N° of Pens	N° (%) of Pens with Intervention	N° of Piglets Evaluated	N° of Piglets Removed due to Tail Biting	% of Removals
Nursery	NTD	28	14 (50)	384	0	0
	TD	28	2 (7)	384	0	0
Rearing/finishing	NTD	28	13 (46)	351	4	1.13
	TD	28	0 (0)	357	1	0.28

## Data Availability

Restrictions apply to the availability of data. Data were obtained from Agroceres PIC and are available upon request from the corresponding author with the permission of Agroceres PIC.
